# Green finance and the synergy of ESGGI performance of Chinese companies: Does green concern matter?

**DOI:** 10.1371/journal.pone.0295706

**Published:** 2023-12-08

**Authors:** Lanlan Liu, Guomin Song

**Affiliations:** 1 School of Accounting, Shandong Women’s University, Jinan, Shandong Province, People’s Republic of China; 2 Postgraduate Studies Unit, College of Business, Universiti Utara Malaysia, Sintok, Malaysia; Universiti Teknologi MARA, MALAYSIA

## Abstract

This study aims to investigate green finance’s impact on the synergy between ESG and green innovation (ESGGI) performance and examine the potential influence played by stakeholders’ green concerns on this impact. Hence, we calculated the synergy of ESGGI performance based on the entropy method and the coupled coordination degree model and conducted regression analyses on the data of 1143 Chinese companies from 2012 to 2020. The results reveal a remarkable inverted U-shaped relationship between green finance and the synergy of ESGGI performance. Additionally, the green concerns of the government and the media can efficiently moderate green finance’s impact on the synergy of ESGGI performance. Further tests show that green finance’s impact on the synergy of ESGGI performance of SOEs, HPEs, and HTEs is more significant. This paper demonstrates an association between green finance and the synergy of ESGGI performance, which yields new insights for companies to implement green transformation and sustainable development.

## 1. Introduction

In accordance with the Sixth Assessment Report (AR6) issued by the Intergovernmental Panel on Climate Change (IPCC) in March 2023, there was a 1.1°C rise in global surface temperature in 2011–2020 as compared to 1850–1900. According to the statistics released by the website of MSCI Net Zero Tracker, 194 countries have proposed carbon reduction targets as of October 20, 2023, of which 116 countries have proposed net-zero emission targets. It is evident that countries’ recognition of reducing carbon emissions and realizing sustainable development has reached a new and unprecedented level. The growing social awareness of climate change has made sustainable development a hot topic of conversation [1].

Luo & Tang (2022) [2] argued that climate change will have profound and irreversible impacts on business. Companies should align their business strategies with the sustainable development goals (SDGs) [3]. Green transformation has become an essential means for sustainable development [4], and green innovation (GI) is the primary approach for facilitating this transformation [5]. Arguably, companies’ GI capacity is crucial in fostering economic growth and achieving high-quality and sustainable development. Meanwhile, environmental, social, and governance (ESG) engagement is a strategic investment for companies. Carrying out social responsibility practices [6] and disclosing environmental information [7] all contribute to enhancing companies’ financial performance and help them benefit from engaging in social responsibility activities with external stakeholders [8]. It has been revealed that ESG not only enhances corporate financial performance [9] but also reflects a greener approach to corporate development [10]. Therefore, conducting ESG and GI activities are both vital initiatives for companies to achieve green and sustainable development.

The Chinese government proposed a carbon peaking and carbon neutrality goal at the 75th United Nations General Assembly and has formulated a series of initiatives to commit to the accomplishment of the goal of green, low-carbon, and sustainable development. In the context of low-carbon strategy and green transformation of national economy, green finance is a crucial strategy to serve companies’ GI activities [11]. Green finance has both market-oriented environmental regulation characteristics and resource allocation functions of the financial sector [12], which can guide capital flows, enhance resource allocation levels, and promote green development [13].

Currently, there is no agreement among academics as to how green finance affects GI. Scholars have held both facilitator and inhibitionist views. According to the facilitation perspective, green finance can encourage GI. When companies encounter high finance constraints, it will inhibit their GI ability, while green financing initiatives can significantly ease the financial limitations and thus incentivize companies to engage in GI [13,14]. Flammer (2021) found that the issuance of green financial instruments such as green bonds directly promotes the increase of companies’ GI activities [15]. In particular, the setting up of green finance reform and innovation pilot zones is beneficial to foster companies’ GI in the pilot zones [16,17]. According to the inhibitory perspective, companies’ GI is not aided by green finance. Green finance policies have a disincentive effect on the inflow of external capital into innovative fields, and the profitability of banks makes the existing credit policies discourage companies’ innovative activities [18]. Mandatory green insurance policies are detrimental to GI [19]. Some scholars have also taken a national macro perspective and found that the long-term effects of green finance on GI are detrimental in non-emerging nations [20].

Previous studies have revealed that green finance motivates companies to adopt proactive environmental responsibility measures [21]. Therefore, with the advancement of green development and green finance policies, more and more financial institutions require the incorporation of corporate ESG performance into the credit process. Green financial system requires companies to disclose ESG information, which is of great significance for companies to engage in environmental management [12]. Green finance policies have been demonstrated to be effective at enhancing companies’ ESG performance [10,12], especially those with lower financing constraints and state-owned companies [22].

Realizing the synergy of companies’ ESGGI performance is to make both sides reach an enhanced state at the same time, which is more helpful to the green sustainable development of companies. From recent studies on ESG performance and GI, most scholars believe that GI can be promoted through companies’ ESG performance [23,24], that GI contributes to companies’ ESG scores [25], and that there is a causal relationship between the two [26]. In contrast to the views expressed above, Cohen et al. (2020) [27] found that oil, gas and energy production companies are the main innovators of green patents in the US, however, these companies have relatively low ESG scores.

Some scholars have also explored related synergies in the economic field, such as Wu & Hu (2020) [28] investigated how government subsidies and slack resources could work together to spur innovation in green technologies. Wang et al. (2023) [21] established a coupling coordination degree model and found that the synergy between green finance and GI efficiency in Chinese provinces was low and had strong regional heterogeneity. According to Ouyang et al. (2022) [29], GI would be significantly impacted in a U-shaped curve by the strategic synergy of local and community environmental regulation. From the perspective of industrial structure, Zhang et al. (2022) [30] explored the quantification of synergy of economic, energy, environmental, and social objectives. He et al. (2023) [31] adopted a synergy model to evaluate the synergy of regional carbon and pollution reduction and discovered that green finance policies can help to strengthen this synergy.

The majority of current research, however, concentrate on how green finance affects GI, ESG, or the linked impacts of ESG and GI, but rarely focus on the synergy of ESGGI performance and how green finance affects this synergy. As a financial policy innovation, how does green finance affect the synergy of ESGGI performance of Chinese companies? Do stakeholders’ green concerns enhance green finance’s impact on this synergy? To address the above issues, this paper first measures the synergy of Chinese companies’ ESGGI performance, empirically examines green finance’s impact on this synergy, and then verifies the role played by stakeholders’ green concerns in this impact.

Based on this, there are three main possible marginal contributions to this paper: First, existing studies pay less attention to the synergy of ESGGI performance, this paper builds a synergy model and measures the synergy of ESGGI performance, which provides positive evidence of the synergistic enhancement of ESGGI performance. Second, given that few existing studies have explored green finance’s impact on the synergy of ESGGI performance, this paper empirically investigates this impact using data from Chinese companies. This paper offers an insightful guide for how companies can utilize green finance policies to help synergize their ESG and GI performance. Third, considering that companies cannot develop without the support of stakeholders, this paper verifies the role of stakeholders’ green concern in the process of green finance in influencing the synergy of ESGGI performance from the media and government perspectives. This study will contribute to further enriching the applied research on ESGGI synergy and green finance’s policy effects and will assist in the green transformation and sustainable development of companies.

The remainder of the paper is organized as follows: The theoretical analysis and research hypotheses are presented in Section 2, followed by the research design in Section 3, the empirical results in Section 4, the discussion in Section 5, and the conclusions and recommendations in Section 6.

## 2. Theoretical analysis and research hypothesis

### 2.1 Green finance and the synergy of ESGGI performance

Resource base theory considers that knowledge, capital, and technology are all resources of a company [32]. Ozdemir et al. (2023) [33] argued that resources are heterogeneous and capable of forming companies’ competitive advantages. Companies often encounter challenges such as high financing costs and an inadequate supply of capital for innovation when pursuing green transformation [34]. These challenges are obviously insoluble by relying only on internal financing. Green finance is a financial resource allocation activity with environmental protection as the primary goal, mainly providing financial assistance for green economic activities [35], which can help companies relieve financing constraints and decrease financing cost, thus guaranteeing the green project’s smooth execution [36]. Thus, green finance is arguably a powerful resource for companies to achieve green and sustainable development.

Chouaibi, et al. (2022) [8] argued that ESG performance can be instrumental in enhancing financial performance and that GI mediates remarkably in this process. Wang et al. (2023) [21] concluded that the implementation of green finance policies motivates companies to adopt positive environmental responsibility measures. Some scholars have concluded that green finance is instrumental not only in enhancing companies’ ESG performance [10,12], but also in promoting GI [37,38]. Particularly, it is more helpful to enhance companies’ GI in the green finance reform and innovation pilot zones [16,17,39]. Hence, from this perspective, green finance may synergistically enhance companies’ ESGGI performance.

However, some academics have asserted that the release of ESG information by companies may induce greenwashing behavior [40]. Companies cooperate with nearby companies on social responsibility activities with the aim of obtaining financing or creating a favorable image [41]. According to Yu et al. (2021) [13], green finance policies cannot alleviate SMEs’ financing constraints, which restrains their GI capabilities. Therefore, there may be the possibility that companies, in order to obtain green finance financial support, increase ESG disclosure and conduct strategic GI to obtain green finance support, and thus the possibility that green finance enhances the synergy of ESGGI performance in the early stage of green finance development. However, with the deepening of the green finance policy, the one-sided pursuit of quantitative ESGGI performance will shift to the pursuit of quality, and the possibility of green finance inhibiting the synergy of corporate ESGGI performance will also emerge. On this basis, Hypothesis 1 (H1) is proposed.

**H1:** The impact of green finance on the synergy of ESGGI performance is inverted U-shaped.

### 2.2 Green finance, green concern and the synergy of ESGGI performance

Stakeholder theory suggests that shareholders, employees, suppliers, customers, financial institutions, government, and communities are all companies’ stakeholders [42]. Jayaraman et al. (2023) [43] argued that stakeholders’ concerns about the environment and pollution motivate companies to engage in green innovations. Chouaibi et al. (2022) [8] concluded that companies engaging in ESG activities could improve their connections with stakeholders such as shareholders, customers, and communities. Meanwhile, as the digital economy and information technology develop rapidly, the influence of external stakeholders on companies will become increasingly relevant.

#### 2.2.1 Media’s green concern

The media serves a dual function of disseminating information and providing corporate governance in the age of the digital economy, and it is a powerful medium for stakeholders to recognize companies [44]. The public’s green concern for companies is a kind of external supervision, and media’s concern can urge companies to take proactive action to address environmental issues. The public’s green concern is also an incentive for GI, and external pressure can boost companies’ green transformation [45,46]. Studies indicate that the media acts as a disseminator of information. Luo et al. (2019) [44] verified that an increase in the level of negative media coverage mitigates the negative effect of environmental disclosure quality on the cost of debt financing. He et al. (2022), in their study of GI in China’s heavy pollution industry, found that media concern can motivate companies to fulfill their responsibilities to the environment and thus carry out GI activities [47]. Geng et al. (2023) [46] revealed that media favorability enhances public environmental concern’s effect on GI in Chinese heavy pollution companies.

The media’s green concern can raise companies’ awareness of environmental issues and promote their development and application of green technologies [48]. Meanwhile, ESG has attracted considerable attention from all facets of society as the public has become increasingly concerned about green development and sustainable development [49]. Media’s concern can also have a significant impact on stakeholders’ investments, such as financial institutions, in companies’ initiatives to innovate [50], because media’s green concern to companies can increase the transparency of companies’ information, which in turn influences the decision-making of stakeholders such as financial institutions. Therefore, the media’s green concern for companies can strengthen the influence of green finance on the synergy of ESGGI performance. On this basis, Hypothesis 2 (H2) is proposed.

**H2:** The media’s green concern can strengthen green finance’s influence on the synergy of ESGGI performance.

#### 2.2.2 Government’s green concern

As a stakeholder of companies, the government’s granting of green subsidies to companies is an obvious mean to assist their green development, and reflects the government’s concern for companies’ green development. Meanwhile, according to the resource base theory, the green subsidies given to companies by the government form their resources, and the resources that companies possess enable them to gain a competitive advantage [33]. As GI has the dual externalities of environmental protection and knowledge spillover [48,51], it leads to a lack of companies’ investment willingness [52]. Shao & Chen (2022) also revealed that government’s concern and support for companies’ green development can facilitate the enhancement of companies’ knowledge base and technology accumulation and that government environmental subsidies are one of the most conducive means to address the environmental protection externalities of GI [52].

In practice, the government mainly supports and encourages companies’ green activities through green subsidies [53]. The government’s green concern to companies, especially the green subsidies to companies, may send the market a optimistic signal about the green development of companies [54], thus increasing the possibility of companies to obtain green financing from financial institutions [55]. From the existing studies, some scholars have found that companies’ acquisition of subsidies can assist them in obtaining subsequent financing [55], which in turn has an impact on their activities. Deng et al. (2022) identified that the association between innovation and environmental performance of Chinese energy-intensive companies is moderated positively by government subsidies, as they can mitigate companies’ financial pressures and financial risks [54]. Therefore, the government’s green concern for companies can enhance green finance’s influence on the synergy of ESGGI performance. On this basis, Hypothesis 3 (H3) is proposed.

H3: The Government’s green concern can enhance green finance’s influence on the synergy of ESGGI performance.

**Fig 1:**
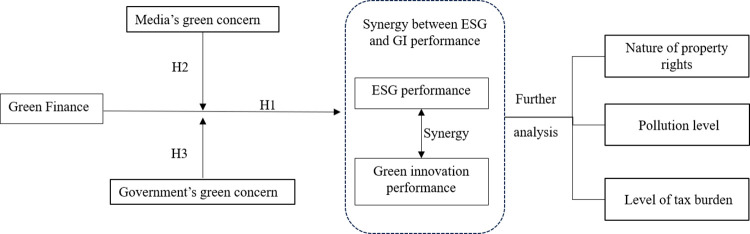
Theoretical framework.

This paper adheres to the theoretical framework described in Fig 1.

## 3. Research design

### 3.1 Sample and data source

The sample of this paper is 1,143 A-share listed companies in China after excluding ST, *ST, and financial industries as well as companies with missing main variables. Chinese Research Data Services (CNRDS) Platform provides companies’ GI data. The Bloomberg database provides information on ESG performance. Data on green finance is sourced from the China Insurance Yearbook, provincial statistical yearbooks, and the China Statistical Yearbook. The China Stock Market & Accounting Research (CSMAR) Database provides information on other variables.

### 3.2 Definition of variables

#### 3.2.1 Dependent variable

The dependent variable is the synergy of ESGGI performance (*Desg_gia*), which is represented by the synergy of companies’ ESG and GI performance as measured by the synergy model. In particular, the measures of ESG and GI performance are as follows.

*Measurement indicators of ESG performance*. Considering the comprehensiveness, rationality and accessibility of indicator selection, and drawing on the practice of Ge et al. (2022) [56], this paper selects Bloomberg’s ESG data to measure the indicators of corporate ESG performance by the three dimensions of Environmental, Social and Governance indicators.

*Measurement indicators of GI performance*. Drawing on Fang & Shao (2022) [5], GI performance (*gia*) is measured using companies’ green patent applications.

#### 3.2.2 Independent variable

Draws on the practice of Liu et al. (2023) [57], green finance (*gfi*) is measured by the regional green finance development index. To ensure the rationality and availability of the index, the index consists of four dimensional indicators, namely, green credit (Interest expenditures of six high energy-consuming industrial industries/Total industrial interest expenditures), green investment (Investment in environmental pollution control/GDP), green insurance (Agricultural insurance income/Gross agricultural output), and government green support (Fiscal environmental protection expenditures/Fiscal general budget expenditures), which are measured by entropy value method.

#### 3.2.3 Moderating variable

Green concern is defined by the green concerns of the media and government, respectively. Among them, the media’s green concern (*gatt*) is represented by the ratio of the number of negative environmental reports of a company to the number of negative environmental reports of the sample companies in the year. The government’s green concern (*gsub*) is indicated by the ratio of the amount of green subsidies received by a company from the government in the year to its total profit in the year.

#### 3.2.4 Control variables

Drawing on Ge et al. (2022) [56], Zhang & Jin (2022) [58], and Tang (2022) [59], the control variables include financial leverage (*lev*), growth capacity (*gro*), firm value (*tbq*), nature of property rights (*pro*), board size (*bds*), duality (*dual*), equity structure (*lcr*), and internal control effectiveness (*linc*).

### 3.3 Method and model

#### 3.3.1 Measurement method of the synergy of ESGGI performance

Due to the large number of corporate ESG performance indicators, this paper applies the entropy weighting method when measuring the weights of the ESG indicators.

#### Step1: Standardize the raw ESG performance data


$${esg}_{ijt}^{,}=\frac{{esg}_{ijt}-min({esg}_{ijt})}{max({esg}_{ijt})-min({esg}_{ijt})}$$ Eq (1)

$$\mathrm{or}\;{esg}_{ijt}^{,}=\frac{max\left({esg}_{ijt}\right)-{esg}_{ijt}}{max({esg}_{ijt})-min({esg}_{ijt})}$$ Eq (2)

Where *esg*_*ijt*_ represents the value of indicator *j* of firm *i* in year *t*; if the indicator j is positive, Eq (1) is used, and if it is negative, Eq (2) is used.

#### Step 2: Calculate the weight of the indicators


$${Pesg}_{ijt}=\frac{{esg}_{ijt}^{,}}{\sum {esg}_{ijt}}$$ Eq (3)

Where *Pesg*_*ijt*_ represents the weight of indicator *j* of firm *i* in year *t*.

#### Step 3: Calculate the information entropy of indicators


$${Eesg}_{ijt}=-\frac{1}{ln(m)}\sum \left[P{esg}_{ijt}\times \ln\left(P{esg}_{ijt}\right)\right]$$ Eq (4)

Where *Eesg*_*ijt*_ is the information entropy corresponding to indicator *j* of firm *i* in year *t*; in this paper, we use the data from 2012 to 2020, so m takes the value of 9.

#### Step 4: Calculate indicator weights


$${Desg}_{ijt}=1-{Eesg}_{ijt}$$ Eq (5)

Where *Desg*_*ijt*_ is the coefficient of variation for indicator *j*.

$${Wesg}_{ijt}=\frac{{Desg}_{ijt}}{\sum D{esg}_{ijt}}$$ Eq (6)

Where *Wesg*_*ijt*_ is the weight of indicator *j*.

#### Step 5: Calculate the composite score of the indicators


$${Sesg}_{ijt}={Wesg}_{ijt}*{esg}_{ijt}$$ Eq (7)

The index of the quantitative degree of the combination of benefits between the systems of corporate ESGGI performance is reflected by the coordination coefficient, so that the comprehensive evaluation function of the performance level of corporate ESG performance subsystems at time t is *f*(*x*, *t*), which is calculated as follows:

$$f(x,t)=\sum {Wesg}_{ijt}\times {esg}_{ijt}^{,}$$ Eq (8)

Similarly, let the comprehensive evaluation function of the performance level of the GI performance system at time *t* be *f*(*y*, *t*).

The coordination coefficient is subsequently calculated with the following formula, utilizing the capacity coupling coefficient model in physics:

$$CC=\frac{2\sqrt{f(x,t)\times f(y,t)}}{f(x,t)+f(y,t)}$$ Eq (9)

To precisely reflect the synergy of ESGGI performance, the synergy degree is introduced to measure the degree of synergistic development between the two, and the formula is as follows:

$$D=\sqrt{CC\times T}$$ Eq (10)

In Eq (10), T=ρf(x,t)+σf(y,t), *ρ* and *σ* are weights. In this paper, we believe that corporate ESG performance is as important as GI performance, so we make *ρ* = *σ* = 0.5, and substitute it into Eq (10) to obtain:

$$D=\sqrt[{4}]{f(x,t)\times f(y,t)}$$ Eq (11)

In Eq (11), 0 ≤ *D* ≤ 1, when *D* tends to 1, the synergy of ESGGI performance is larger, and the two have reached the degree of effective synergistic development. Conversely, the synergy between the two is smaller.

#### 3.3.2 Model construction

In order to test green finance’s influence on the synergy of ESGGI performance, this paper constructs the following model:

$$Des{g\_gia}_{it}=\alpha_{0}+\alpha_{1}\times {gfisq}_{it}+\alpha_{2}\times {gfi}_{it}+\alpha_{3}\times {CONTROLS}_{it}+\mu_{i}+\delta_{t}+\theta_{i}+\pi_{i}+\varepsilon_{it1}$$ Model (1)

Where *Desg*_*gia*_*it*_ is the synergy of ESGGI performance; *i* is individual companies, *t* is time; *gfi*_*it*_ is green finance; *CONTROLS*_*it*_ symbolizes the control variables; *μ*_*i*_, *δ*_*t*_, *θ*_*i*_, *π*_*i*_ denote control firm, year, industry and region fixed effects, respectively; and *ε*_*it*1_ is the residual term.

In order to test the moderating role played by the green concern of the media and the government for companies on the above effect, this paper also establishes the following moderating effect models:

$$Des{g\_gia}_{it}=\beta_{0}+\beta_{1}\times {gfisq}_{it}+\beta_{2}\times {gfi}_{it}+\beta_{3}\times {gfisq}_{it}\times {gatt}_{it}+\beta_{4}\times {gfi}_{it}\times {gatt}_{it}+\beta_{5}\times {gatt}_{it}+\beta_{6}\times {CONTROLS}_{it}+\mu_{i}+\delta_{t}+\theta_{i}+\pi_{i}+\varepsilon_{it2}$$ Model (2)

$$Des{g\_gia}_{it}=\gamma_{0}+\gamma_{1}\times {gfisq}_{it}+\gamma_{2}\times {gfi}_{it}+\gamma_{3}\times {gfisq}_{it}\times {gsub}_{it}+\gamma_{4}\times {gfi}_{it}\times {gsub}_{it}+\gamma_{5}\times {gsub}_{it}+\gamma_{6}\times {CONTROLS}_{it}+\mu_{i}+\delta_{t}+\theta_{i}+\pi_{i}+\varepsilon_{it3}$$ Model (3)

Where *gatt*_*it*_ is media green concern; *gsub*_*it*_ is government green concern; *ε*_*it*2_, *ε*_*it*3_ are the residual terms; the definitions of the remaining variables in Model (2) and Model (3) are the same as in Model (1).

## 4. Empirical results

### 4.1 Descriptive statistics

The descriptive statistics findings for the primary variables are shown in Table 1. The maximum value of *Desg_gia* of the sample companies is 0.9016, the minimum value is 0.0010, and the mean value is 0.0843, indicating that the sample companies’ synergy of ESGGI performance fluctuates greatly and the overall synergy is low.

**Table 1:** Descriptive statistics results.

Variable	Obs	Mean	Std.Dev.	Min	Max
Desg_gia	8,876	0.0843	0.1153	0.0010	0.9016
gfi	8,876	0.2912	0.1705	0.0710	0.8390
gatt	8,876	0.0005	0.0016	0.0000	0.0530
gsub	8,876	0.0086	0.2744	-1.5460	19.6527
lev	8,876	0.4777	0.2004	0.0493	0.9796
gro	8,876	0.3905	1.0861	-0.7891	10.2879
tbq	8,876	1.0075	0.3484	0.5245	3.4067
pro	8,876	0.5170	0.4997	0.0000	1.0000
bds	8,876	2.2868	0.1828	1.3863	2.9444
dual	8,876	0.2022	0.4017	0.0000	1.0000
lcr	8,876	4.0590	0.2927	2.2802	4.6265
linc	8,876	3.6047	0.1987	1.4748	3.9977

The maximum value of *gfi* is 0.8390, the minimum value is just 0.0710, and the mean value is 0.2912, meaning that the development of green finance is still in the preliminary stage and is not balanced across different regions. The minimum value of *gatt* is 0, the maximum value is 0.0530, and the mean value is 0.0005, showing that the proportion of negative environmental reports of the sample companies is relatively low. The minimum value of *gsub* is -1.5460, the maximum value is 19.6527, and the mean value is 0.0086, revealing that the sample companies’ green subsidy level from the government varies greatly.

Table 2 shows the synergy of ESGGI performance of the sample companies according to their regions. As can be seen, the maximum value of *Desg_gia* for the sample companies during the period of 2012–2020 occurs in Beijing at 0.9016, and the sub-maximum value of *Desg_gia* occurs in Guangdong at 0.8738. The maximum values of *Desg_gia* for the sample companies in the provinces of Hunan, Anhui, Xinjiang, Henan, Shandong, Sichuan, and Shanxi are all greater than 0.6. However, it can also be seen from the statistics that the *Desg_gia* values of the sample companies in each province fluctuates greatly and the mean value is small overall, indicating that the synergy of ESGGI performance across Chinese provinces is still fairly low.

**Table 2:** Descriptive statistics of synergy of ESGGI performance by region.

Province	Obs	Mean	Std.Dev.	Min	Max
Beijing	1,073	0.0883	0.1343	0.0077	0.9016
Tianjin	156	0.0368	0.0486	0.0176	0.2801
Hebei	176	0.0948	0.1233	0.0168	0.5010
Shanxi	169	0.0791	0.1129	0.0198	0.6056
Inner Mongolia	101	0.0726	0.0930	0.0162	0.3391
Liaoning	210	0.0772	0.1095	0.0102	0.5504
Jilin	100	0.0357	0.0469	0.0010	0.2327
Heilongjiang	112	0.0503	0.0710	0.0162	0.3810
Shanghai	769	0.0680	0.1028	0.0101	0.5621
Jiangsu	543	0.0843	0.1018	0.0160	0.5330
Zhejiang	888	0.0739	0.0973	0.0010	0.4651
Anhui	306	0.1348	0.1375	0.0165	0.6618
Fujian	477	0.0643	0.0894	0.0165	0.5404
Jiangxi	152	0.0768	0.0940	0.0010	0.3772
Shandong	492	0.1112	0.1307	0.0135	0.6373
Henan	312	0.1023	0.1169	0.0010	0.6455
Hubei	247	0.0823	0.0992	0.0145	0.3930
Hunan	222	0.0730	0.1217	0.0122	0.6802
Guangdong	1,208	0.1064	0.1377	0.0118	0.8738
Guangxi	67	0.0832	0.1040	0.0186	0.3677
Hainan	56	0.0258	0.0261	0.0186	0.2168
Chongqing	140	0.0610	0.0919	0.0165	0.3939
Sichuan	271	0.0813	0.1094	0.0131	0.6134
Guizhou	89	0.0582	0.0686	0.0177	0.2745
Yunnan	139	0.0888	0.0990	0.0160	0.4202
Shaanxi	108	0.1036	0.1193	0.0160	0.5275
Gansu	58	0.0798	0.0991	0.0177	0.3524
Qinghai	66	0.0398	0.0563	0.0160	0.2818
Ningxia	34	0.0560	0.0713	0.0169	0.2639
Xinjiang	135	0.0761	0.1205	0.0141	0.6476

All variables’ VIF values fall under the threshold of 10, and the mean VIF value is 1.11, suggesting that there is no significant covariance problem among the variables.

### 4.2 Basic test

According to the results of the Hausman test, this paper elects the fixed effect model for regression analysis. Columns (1) and (2) of Table 3 illustrate green finance’s impact on the synergy of ESGGI performance. The results show that the regression coefficients for *gfisq* are remarkably negative at the 1% significance level regardless of the inclusion of control variables, while the regression coefficients for *gfi* are notably positive at the 5% significance level, and the results pass the Utest check. According to the findings, there is a significant inverted U-shaped curve between green finance and the synergy of ESGGI performance.

**Table 3:** Basic and moderating effect test results.

Variable	Model (1)		Model (2)		Model (3)	
	(1)	(2)	(3)	(4)	(5)	(6)
gfisq	-0.154***	-0.159***	-0.155***	-0.161***	-0.168***	-0.171***
	(-2.642)	(-2.721)	(-2.659)	(-2.744)	(-2.870)	(-2.915)
gfi	0.184**	0.192**	0.189**	0.198**	0.196**	0.202**
	(2.133)	(2.215)	(2.186)	(2.273)	(2.263)	(2.319)
gatt			0.937	1.076		
			(1.135)	(1.302)		
gatt* gfisq			-29.517*	-29.364*		
			(-1.733)	(-1.724)		
gatt* gfi			25.588*	25.039*		
			(1.903)	(1.863)		
gsub					-0.003	-0.003
					(-0.339)	(-0.403)
gsub* gfisq					-0.598**	-0.542*
					(-2.099)	(-1.901)
gsub* gfi					0.435*	0.386
					(1.693)	(1.503)
_cons	-0.031	-0.090	-0.030	-0.088	-0.033	-0.089
	(-0.406)	(-1.084)	(-0.393)	(-1.057)	(-0.429)	(-1.065)
CONTROLS	N	Y	N	Y	N	Y
Firm FE	Y	Y	Y	Y	Y	Y
Year FE	Y	Y	Y	Y	Y	Y
Industry FE	Y	Y	Y	Y	Y	Y
Region FE	Y	Y	Y	Y	Y	Y
N	8876	8876	8876	8876	8876	8876
R^2^	0.025	0.028	0.027	0.029	0.026	0.029

Note: T-values in parentheses; *, **, ***, respectively, indicate the significance levels of 10%, 5%, and 1%.

The basic test results demonstrate that green finance can empower the synergistic enhancement of ESGGI performance when the green finance development level is within a certain threshold. When the green finance development level exceeds the threshold, green finance’s impact on the synergy of ESGGI performance shows an inhibitory trend. These results verify H1.

### 4.3 Moderating effect test

This paper decentralizes green concern, green finance, and other associated factors prior to assessing the moderating role of green concern with the goal of eliminating any potential multicollinearity issues between the interaction term and the independent variables. The results of the moderating effect of green concern on green finance affecting the synergy of ESGGI performance are listed in Table 3.

As seen in columns (3) and (4) of Table 3, with the inclusion of media green concern (*gatt*), its interaction with the squared term of green finance (gatt*gfisq), and its interaction with green finance (gatt*gfi), green finance’s influence on the synergy of ESGGI performance is unchanged. Whereas, the interaction term gatt*gfisq is negatively related to *Desg_gia* at the 10% significance level. This result suggests that the media’s green concern for companies enhances green finance’s influence on the synergy of ESGGI performance. It also implies that the more negative reports on corporate environmental protection there are, the stronger green finance’s influence on the synergy of ESGGI performance is. This result verifies H2.

As shown in columns (5) and (6) of Table 3, with the inclusion of government green concern (*gsub*), its interaction with the squared term of green finance (gsub*gfisq), and its interaction with green finance (gsub*gfi), green finance’s influence on the synergy of ESGGI performance are also unchanged. At the same time, the interaction term gsub*gfisq is negatively related to *Desg_gia*. This result suggests that the government’s green concern enhances green finance’s influence on the synergy of ESGGI performance and that the larger the contribution of government green subsidies to profits, the stronger green finance’s influence on the synergy of ESGGI performance is. This result verifies H3.

### 4.4 Endogeneity test

In the previous empirical model, there may be a certain endogenous effect of green finance and the synergy of ESGGI performance. With a view to minimizing this effect, this paper draws on Li & Shen (2019) [60] and uses the number of banks in the Republic of China (ROC) period as an instrumental variable of green finance to perform 2SLS estimation on Model (1). The results are summarized in Table 4.

**Table 4:** 2SLS regression results.

Variable	First-stage		Second-stage
	gfisq	gfi	Desg_gia
banksq	-0.000***	-0.000***	
	(-17.967)	(-23.323)	
bank	0.002***	0.005***	
	(27.231)	(33.758)	
gfisq			-3.175**
			(-2.240)
gfi			1.274**
			(1.998)
_cons	-0.010	0.080***	-0.242***
	(-0.859)	(3.463)	(-3.249)
CONTROLS	Y	Y	Y
Year FE	Y	Y	Y
N	7385	7385	7385
R^2^	0.349	0.385	-

Note: T-values in parentheses; *, **, ***, respectively, indicate the significance levels of 10%, 5%, and 1%.

The first-stage regression result shows that the number of banks is highly correlated with green finance and their F-statistics are all over 10, which indicates that there is no weak instrumental variable problem. The second-stage regression result reveals that green finance still has a substantial inverted U-shaped impact on the synergy of ESGGI performance.

### 4.5 Robustness test

This paper conducts robustness tests on the aforementioned test results using the lagged one-period (*L*.*Desg_gia*) and lagged two-period (*L2*.*Desg_gia*) data of synergy of ESGGI performance, as well as lagged one-period data of green finance. Table 5 displays the test results.

**Table 5:** Robustness test results.

Variable	(1)	(2)	(3)
	L.Desg_gia	L2.Desg_gia	Desg_gia
gfisq	-0.175***	-0.295***	
	(-2.766)	(-4.076)	
gfi	0.209**	0.426***	
	(2.187)	(3.809)	
L.gfisq			-0.170***
			(-2.917)
L.gfi			0.191**
			(2.420)
_cons	-0.002	-0.020	-0.058
	(-0.018)	(-0.190)	(-0.670)
CONTROLS	Y	Y	Y
Firm FE	Y	Y	Y
Year FE	Y	Y	Y
Industry FE	Y	Y	Y
Region FE	Y	Y	Y
N	7790	6687	7791
R^2^	0.016	0.016	0.030

Note: T-values in parentheses; *, **, ***, respectively, indicate the significance levels of 10%, 5%, and 1%.

According to the findings in columns (1) and (2), *gfisq* is negatively associated with the lagged one-period data of the synergy of ESGGI performance (*L*.*Desg_gia*) at the 5% significance level, and is negatively associated with the lagged two-period data of the synergy of ESGGI performance (*L2*.*Desg_gia*) at the 1% significance level. Meanwhile, *gfi* is all significantly positively correlated with the dependent variable. These findings imply that the robustness of green finance’s inverted U-shaped effect on the synergy of ESGGI performance.

Further from the test results of the lagged one-period data of green finance, the squared term lagged one-period of green finance (*L*.*gfisq*) is negatively correlated with *Desg_gia* at the 1% significance level, and the lagged one-period of green finance (*L*.*gfi*) is positively correlated with *Desg_gia* at the 5% significance level. The robustness test findings further validate the previous conclusions.

### 4.6 Further analysis

This paper divides sample companies into three groups: state-owned companies (SOEs) versus non-state-owned companies (NSOEs), heavily polluting companies (HPEs) versus non-heavily polluting companies (NHPEs), and high-tax-burdening companies (HTEs) versus low-tax-burdening companies (LTEs) to examine the heterogeneity of green finance’s impact on ESGGI performance.

#### 4.6.1 Further analysis based on the nature of property rights

In line with the findings in Table 6’s columns (1)-(2), *gfisq* has a substantial negative impact on *Desg_gia* of SOEs, and *gfi* has a substantial positive impact on *Desg_gia* of SOEs, and the result passes the Utest check. This indicates that green finance has a significant inverted U-shaped effect on the synergy of ESGGI performance of SOEs. Differently from SOEs, *gfi* and *gfisq* do not influence *Desg_gia* of NSOEs.

**Table 6:** Test results for further analysis.

Variable	SOEs	NSOEs	HPEs	NHPEs	HTEs	LTEs
	(1)	(2)	(3)	(4)	(5)	(6)
	Desg_gia	Desg_gia	Desg_gia	Desg_gia	Desg_gia	Desg_gia
gfisq	-0.232***	-0.051	-0.477***	-0.026	-0.245***	-0.065
	(-3.064)	(-0.536)	(-3.405)	(-0.399)	(-2.852)	(-0.706)
gfi	0.304***	0.058	0.564***	0.004	0.319**	0.046
	(2.697)	(0.411)	(2.799)	(0.046)	(2.497)	(0.339)
_cons	-0.057	0.016	-0.048	-0.245***	-0.053	-0.343***
	(-0.838)	(0.121)	(-0.545)	(-2.700)	(-0.720)	(-2.726)
CONTROLS	Y	Y	Y	Y	Y	Y
Firm FE	Y	Y	Y	Y	Y	Y
Year FE	Y	Y	Y	Y	Y	Y
Industry FE	Y	Y	Y	Y	Y	Y
Region FE	Y	Y	Y	Y	Y	Y
N	4589	4287	2308	6568	4185	4691
R^2^	0.033	0.032	0.044	0.032	0.029	0.039

Note: T-values in parentheses; *, **, ***, respectively, indicate the significance levels of 10%, 5%, and 1%.

#### 4.6.2 Further analysis based on pollution level

Considering the results reported in Table 6’s columns (3)-(4) of Table 6, *gfisq* significantly and negatively affects *Desg_gia* of HPEs, whereas *gfi* significantly and positively affects *Desg_gia* of HPEs, and the result also passes the Utest check. This finding implies the synergy of ESGGI performance of HPEs with an inverted U-shaped curve is significantly influenced by green finance. Unlike HPEs, *gfi* and *gfisq* do not have a significant effect on *Desg_gia* of NHPEs.

#### 4.6.3 Further analysis based on the level of tax burden

As shown in results in columns (5)-(6) of Table 6, *gfisq* negatively affects *Desg_gia* of HTEs at 1% level, whereas *gfi* positively affects *Desg_gia* of HTEs at 5% level, and the result passes the Utest check. The result of this test implies that green finance significantly affects the synergy of ESGGI performance of HTEs with an inverted U-shaped curve. Unlike HTEs, *gfi* and *gfisq* also do not have a significant effect on *Desg_gia* of LTEs.

## 5. Discussion

### 5.1 Green finance and the synergy of ESGGI performance

In measuring the synergy of Chinese companies’ ESGGI performance, we find that the synergy of Chinese listed companies varies widely, is low overall, and shows significant regional variations. Similar to this finding, Wang et al. (2023) [21] measured the synergy of green finance and GI and came to the conclusion that the synergy is at a low or basic level in the majority of Chinese regions. The empirical investigation in this paper reveals that the synergy of ESGGI performance is significantly affected nonlinearly by green finance. Similarly, Ouyang et al. (2022) [29] demonstrated that strategic synergy of environmental regulation also has a non-linear impact on companies’ GI efficiency.

There are two possible reasons for this nonlinear effect. First, more and more financial institutions incorporate ESG performance into the credit process, forcing companies to improve their ESG performance. Second, green finance mainly supports the financing of green economic activities [35], and companies may proliferate low-quality GI by pursuing speed and quantity [61], signaling environmental compliance [62], which leads to the possibility that when green finance develops within a certain level, it can enhance the synergy of ESGGI performance. With the continuous improvement of green finance policy and its auditing and evaluation systems, the speculative behavior of companies in carrying out strategic green innovations will be reduced, and thus there is a possibility that green finance will reduce the synergy of ESGGI performance. This conclusion also suggests a direction for financial institutions to advance green financial system and effectively play the guiding role of green capital allocation.

### 5.2 The moderating role of green concern

The paper’s findings also imply that stakeholders’ green concerns can enhance green finance’s influence on the synergy of ESGGI performance.

From the viewpoint of media’s green concern, there are two possible reasons. First, the media plays an influential role in external corporate governance as a mediator of information dissemination [50], given that media concern improves the visualization and transparency of companies’ behaviors, which in turn affects companies’ business decisions [63]. Second, the media’s concern also affects the extent to which stakeholders exert pressure on companies [64]. Especially when companies have external financing needs, the media’s concern can make companies more cautious [65].

From the viewpoint of the government’s green concern, the government’s activities, such as resource allocation and policy and regulation formulation, have a bearing on the companies’ survival and development. Green subsidies are an instrumental way to encourage companies to carry out green activities and promote green development [52], conveying a positive signal of government endorsement [66]. In particular, green subsidies can make it possible to provide companies with the funds they need to invest in green activities, which is a crucial approach to alleviate companies’ financing constraints and enhance their GI ability [52]. Therefore, in conjunction with green finance, it may deepen green finance’s influence on the synergy of ESGGI performance. It also provides a way of thinking for companies to obtain government support, improve ESGGI performance, and realize green transformation.

## 6. Conclusions and recommendations

### 6.1 Conclusions

ESG and GI activities are an effective approach for companies to achieve the goal of sustainable development. This paper attempts to explore green finance’s impact on the synergy of ESGGI performance from the external policy perspective of green finance in order to fill the gap in the research literature on the synergy of ESGGI performance. To this end, this paper empirically examines this issue using data from 1,143 Chinese A-share listed companies from 2012 to 2020.

Our results discover that green finance can notably affect the synergy of corporate ESGGI performance and exhibit an inverted U curve of facilitation followed by inhibition. This finding provides a meaningful reference for companies in the early stages of green transformation to actively seek green finance policy support and synergistically improve corporate ESGGI performance. The government and the media are all vital stakeholders of companies, and their green concern for companies will affect companies’ green activities. As the results of our moderating effect models indicate, the green concern of media and government can remarkably enhance green finance’s impact on the synergy of ESGGI performance. Hence, in the process of green transformation, companies should not only emphasize the green support of financial institutions but also actively seek the concern and support of the government, media, and other stakeholders. Furthermore, we notice in the results of the heterogeneity test that green finance’s impact on the synergy of ESGGI performance of companies shows differentiation. Green finance has a pronounced inverted U-shaped effect on the synergy of ESGGI performance of SOEs, HPEs, and HTEs, while it does not significantly affect the synergy of ESGGI performance of NSOEs, NHPEs, and LTEs. This finding also provides a reference for different types of companies to effectively utilize the advantages of internal and external resources and explore green development.

### 6.2 Policy recommendations

In view of the empirical conclusion that green finance significantly affects the synergy of corporate ESGGI performance, and that the green concerns of stakeholders such as the media and the government can substantially enhance this effect, companies, financial institutions, the government, and the media should collaborate with each other to jointly promote the realization of green and sustainable development goals.

Companies should capitalize on the advantages of green finance policies, proactively engage in ESG and GI activities, and propel companies to transition to green and sustainable development. Financial institutions should take into account the needs of green transformation, continuously develop green financial products, establish a comprehensive green financial audit and evaluation mechanism, and enhance the policy effect of green finance. The green concern of government departments exerts an influential effect on the green transformation of companies, and the green subsidies given by the government have different effects on different types of companies, so differentiated green subsidy strategies should be formulated according to the characteristics of the companies. The media, as an essential medium for the public to concern and supervise the green development of companies, should improve the quality of reporting, and report truthfully, objectively, rationally and responsibly to facilitate companies’ green development.

This paper verifies green finance’s impact on the synergy of ESGGI performance and the role of stakeholders’ green concerns in this impact, basing on the measurement of the synergy of corporate ESGGI performance. This paper not only expands the research on synergy, but also provides a reference for companies to utilize stakeholders’ support to advance the implementation of corporate green transformation strategies.

In spite of the fact that the measurement of ESGGI performance synergy and the empirical tests in this paper are as rigorous as possible, there are still some limitations. First, our research sample has a limitation. Although there are many rating agencies that release ESG score data, considering the authority and availability of the data, our sample is selected only from the sample of Chinese listed companies with Bloomberg ESG scores. Second, for the measurement of ESGGI performance synergy, we selected Bloomberg ESG scores and corporate green patent data to measure respectively, and there may be more suitable indicators in the future. In addition, we focus on the influence of three important stakeholders, namely, financial institutions, government, and media, on the synergy of ESGGI performance, and we may further expand the research on the factors influencing the synergy between ESG and GI performance in the future.
